# Psychopaths Show Enhanced Amygdala Activation during Fear Conditioning

**DOI:** 10.3389/fpsyg.2016.00348

**Published:** 2016-03-10

**Authors:** Douglas H. Schultz, Nicholas L. Balderston, Arielle R. Baskin-Sommers, Christine L. Larson, Fred J. Helmstetter

**Affiliations:** ^1^Department of Psychology, University of Wisconsin–Milwaukee, MilwaukeeWI, USA; ^2^Department of Psychology, Yale University, New HavenCT, USA; ^3^Department of Neurology, Medical College of Wisconsin, MilwaukeeWI, USA

**Keywords:** psychopathy, fear conditioning, anxiety, amygdala, fMRI

## Abstract

Psychopathy is a personality disorder characterized by emotional deficits and a failure to inhibit impulsive behavior and is often subdivided into “primary” and “secondary” psychopathic subtypes. The maladaptive behavior related to primary psychopathy is thought to reflect constitutional “fearlessness,” while the problematic behavior related to secondary psychopathy is motivated by other factors. The fearlessness observed in psychopathy has often been interpreted as reflecting a fundamental deficit in amygdala function, and previous studies have provided support for a low-fear model of psychopathy. However, many of these studies fail to use appropriate screening procedures, use liberal inclusion criteria, or have used unconventional approaches to assay amygdala function. We measured brain activity with BOLD imaging in primary and secondary psychopaths and non-psychopathic control subjects during Pavlovian fear conditioning. In contrast to the low-fear model, we observed normal fear expression in primary psychopaths. Psychopaths also displayed greater differential BOLD activity in the amygdala relative to matched controls. Inverse patterns of activity were observed in the anterior cingulate cortex (ACC) for primary versus secondary psychopaths. Primary psychopaths exhibited a pattern of activity in the dorsal and ventral ACC consistent with enhanced fear expression, while secondary psychopaths exhibited a pattern of activity in these regions consistent with fear inhibition. These results contradict the low-fear model of psychopathy and suggest that the low fear observed for psychopaths in previous studies may be specific to secondary psychopaths.

## Introduction

Psychopathic individuals display antisocial personality traits including deceitfulness, impulsivity, recklessness, lack of remorse, and a general failure to conform to social norms (Cleckley, 1982; American Psychiatric Association, 2013). These symptoms have long been thought to reflect an overall lack of fear resulting from abnormal functioning of the amygdala (Birbaumer et al., 2005; Raine and Yang, 2006; Moul et al., 2012). Consistent with this view, psychopathic offenders show deficits in their ability to use threat-relevant information to inhibit inappropriate approach behavior (Lykken, 1957; Newman and Kosson, 1986; Blair et al., 2004). They also tend to display smaller electrodermal responses to stimuli predicting aversive outcomes (Hare, 1980). Moreover, some studies have shown that psychopaths perform poorly relative to controls during Pavlovian fear conditioning (Birbaumer et al., 2005; Rothemund et al., 2012). The deficits observed in fear conditioning have provided some of the best evidence for the low-fear model of psychopathy.

Although, the low-fear model provides an intuitive framework for understanding the etiology of psychopathic symptoms, there is evidence that multiple genetic, environmental, and developmental factors contribute to the development of psychopathy, and that different types of psychopaths may arise from distinct etiologies. For instance, previous research has shown that psychopathy is a heterogeneous category and that psychopaths can be divided into subgroups based on levels of trait anxiety (Newman et al., 2005; Skeem et al., 2007). Primary psychopaths tend to show low trait anxiety and more closely match the stereotype of the prototypical psychopath. Their symptoms are thought to be inherent and are not an indirect consequence of some other deficit (Lykken, 1957). In contrast, secondary psychopaths tend to show high levels of trait anxiety. Their psychopathy symptoms are thought to arise over the course of development, possibly through the experience of repeated traumatic experience or emotional hyper-reactivity to negative events. While the low fear model of psychopathy intuitively explains the symptoms of primary psychopathy, it is currently unclear whether this model can explain the symptoms of secondary psychopathy. In fact, there is some evidence that primary and secondary psychopaths may respond differently to threat related stimuli (Arnett, 1997). Taken together, these studies suggest that anxiety may affect the pattern of responding in psychopaths during standard Pavlovian conditioning. However, this has yet to be studied.

The purpose of this study was to determine whether the relationship between anxiety and fear acquisition differs in psychopathic inmates and a population of well-matched non-psychopathic offenders from the same institution. According to the low fear model of psychopathy, psychopaths compared to non-psychopaths should show smaller fear responses and less robust amygdala activity during aversive classical conditioning. Additionally, given the distinct etiologies of primary and secondary psychopathy, we predicted that anxiety would affect the neural and behavioral outcomes of fear conditioning.

## Materials and Methods

### Participants

Participants were 66 white male prisoners from a medium security prison in Southern Wisconsin between the ages of 18 and 45. Participants were excluded if they were age 45 or older, currently used psychotropic medication, had clinical diagnoses of schizophrenia, bipolar disorder, or psychosis (not otherwise specified), had contraindications for MR scanning, scored below the 4th grade reading level on achievement tests administered by the Department of Corrections, or had an estimated IQ score of less than 70 on the Shipley Institute of Living Scale (SILS; Zachary and Shipley, 1986). Three participants were dropped due to poor alignment of structural and functional images, six because of movement artifact, one due to claustrophobia, and six because of equipment malfunction. Elements of consent were presented individually to all participants in verbal and written form, according to the Declaration of Helsinki. Participants were also informed that their decision to take part in the project or to refuse would have no influence on their status within the correctional system.

All participants were assessed using file information and a semi-structured interview that lasted approximately 60 min and provided sufficient information to diagnose psychopathy using the Psychopathy Checklist-Revised (PCL-R; Hare and Vertommen, 2003). The PCL-R contains 20 items that are rated 0, 1, or 2 according to the degree to which a characteristic is present: significantly [2], moderately [1], or not at all [0]. Numerous sources have documented the reliability and validity of the PCL-R (Hare et al., 1991; Hare, 1996). To evaluate inter rater reliability, a second rater who was present during interviews provided independent PCL-R ratings for eight inmates. The intraclass correlation coefficient was 0.85. As done in a previous study using this cohort, participants were classified as psychopathic if their PCL-R scores were 30 or greater and non-psychopathic if their PCL-R scores were 20 or less (Motzkin et al., 2011). This provides a clear distinction between these groups (as recommended by Hare and Vertommen, 2003), but precludes correlation approaches using PCL-R scores which would require a normal distribution of scores. The final sample consisted of 19 psychopaths and 31 controls (**Table 1**).

**Table 1:** Demographic information.

Group	*N*	Age	Education	WAS	PCL-R
Psychopath	31	31.63	9.26	12.79	32.12
Control	19	32.16	11.23	10.74	13.22

Examination of psychopathy by anxiety differences was conducted in two ways. First, due to the sample size, we included anxiety as a continuous variable. Second, following the convention of previous studies identifying psychopathic subtypes, psychopaths were subdivided based on a median split of their scores on the Welsh Anxiety Scale (WAS; Hare, 1980; Hare and Vertommen, 2003; Motzkin et al., 2011). The WAS has a strong positive correlation with the State-Trait Anxiety Inventory (Spielgberger et al., 1983), and strong internal consistency (Hale et al., 2004). Thus, in our sample low-anxious (primary) psychopathy was defined as having a PCL-R score of 30 or greater and a WAS score of 13 or less, while high-anxious (secondary) psychopathy was defined as having a PCL-R score of 30 or greater and a WAS score of 14 or greater. Non-psychopathic participants were subdivided into high and low-anxious subgroups using the same WAS scores. When divided into subtypes, the final sample consisted of nine high-anxious (secondary) psychopaths, 10 low-anxious (primary) psychopaths, 11 high-anxious controls, and 20 low-anxious controls.

### Procedure

**FIGURE 1:**
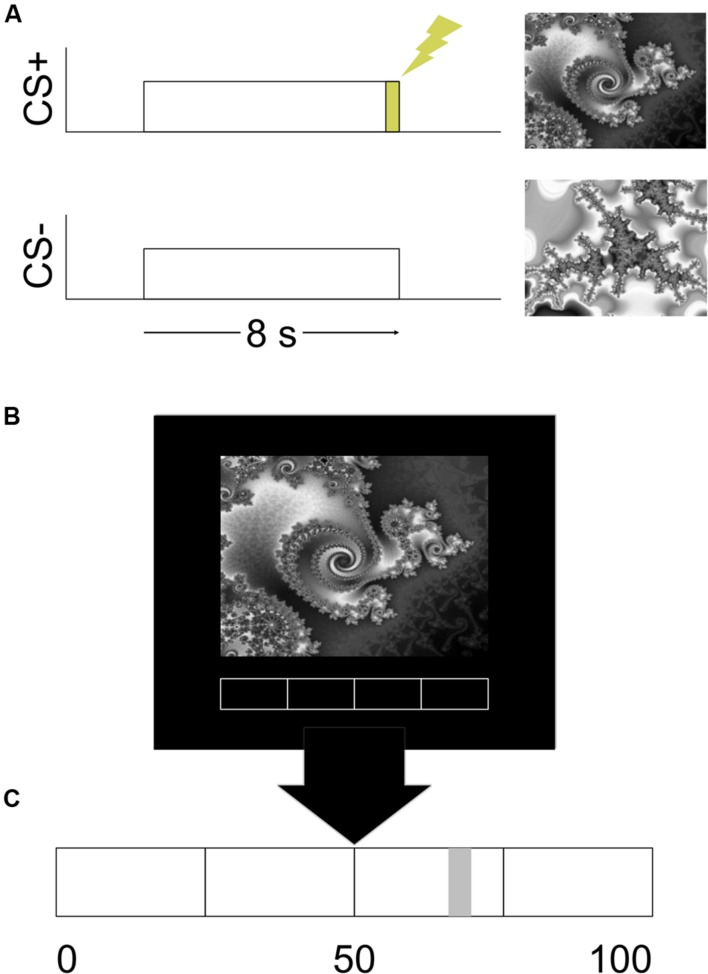
**Design of the experiment. (A)** An example of the stimulus timing for the CS+ and CS- trials. **(B)** An example of the visual display. **(C)** An enlarged example of the rating bar that was present on the bottom of the visual display.

Prior to the day of scanning subjects completed the informed consent and the clinical interview. On the day of the scan, the subject was escorted to the magnetic resonance imaging (MRI) scanner, housed onsite in a mobile trailer, and informed of the procedures. Once the subject was ready to enter the scan room, the operator attached the shock and skin conductance response (SCR) electrodes, performed the shock workup, and instructed the subject on the proper use of the behavioral response device. Prior to the conditioning scan, subjects completed a separate attention task, which is described elsewhere (Larson et al., 2013). During the conditioning scan, subjects received five trials of differential conditioning with visual conditional stimuli (CS) while we recorded BOLD, SCRs, and shock expectancy (see **Figure 1B**; Balderston and Helmstetter, 2010; Schultz et al., 2012, 2013; Balderston et al., 2013).

### Apparatus

#### Visual Stimuli

The Presentation software package (Albany, CA, USA) was used to run the experiment. The visual CS and a rating bar were presented on a mirror attached to the headcoil via a high-resolution screen mounted in the rear of the magnet bore. The CS were two different gray scale fractal images. Images were presented for 8 s (see **Figure 1A**). One image always co-terminated with a 500 ms shock and served as the CS+. The other image was never paired with shock and served as the CS-. The stimuli were presented in a pseudorandom order with the condition that there would be no more than two consecutive stimuli of the same type. The assignment of images to CS type was counterbalanced.

#### UCS Expectancy

Participants reported their expectation of receiving the UCS continuously throughout the study. A rating bar with a scale of 0–100 was always present on the bottom of the visual display (see **Figure 1C**). Participants moved a cursor on the rating bar by button press responses on a fiber-optic response device (Nata Technologies, Coquitlam, BC, Canada) with their right hand. Prior to the start of the experiment participants were given verbal instructions on how to move the cursor. Participants were informed that they could place the cursor anywhere between 0 and 100 on the rating bar. They were instructed to place the cursor at 0 or all the way to the left of the rating bar if they were absolutely certain that they would not receive a presentation of the UCS. Placing the cursor at 50 or in the middle of the rating bar indicated that they were unsure whether or not a presentation of the UCS would occur. A rating of 100 or placing the cursor all the way to the right indicated that they were absolutely certain that they would receive a presentation of the UCS. Participants were instructed to update their ratings continuously throughout the experiment. We did not provide any instructions to the participants regarding any of the programmed experimental contingencies.

#### Electrical Stimulus

A 500 ms presentation of an electrical stimulus served as the UCS. An AC (60 Hz) source (Contact Precision Instruments, Model SHK1, Boston, MA, USA) delivered the stimulation through two surface cup electrodes (Biopac Model EL258-RT, Goleta, CA, USA) filled with electrolytic gel (Signa Gel, Parker Laboratories, Fairfield, NJ, USA) and placed on the skin above the participant’s right tibial nerve above the right medial malleolus. Each participant determined the maximum UCS intensity through a work-up procedure prior to conditioning. The work-up procedure consisted of no more than five presentations of the electrical stimulus. Participants rated the intensity of these presentations on a 0–10 scale. A rating of 0 indicated that they didn’t feel the stimulation. A rating of 10 indicated that the stimulus was painful, but tolerable. The intensity of the electrical stimulation was gradually increased until the participant rated it as a 10. Participants were able to rate the stimulation as above 10 in which case the intensity would be decreased. The intensity that each participant rated as a 10 was what was used in the experiment.

#### Skin Conductance

Galvanic skin responses (GSRs) were recorded for each subject using a pair of disposable adhesive snap electrodes applied to the bottom of the participant’s left foot. The electrodes were connected to magnetically shielded cables and attached to a Biopac Systems skin conductance module (EDA100c-MRI). The GSR signal was amplified and sampled at 100 Hz. This signal was the stored on a laptop computer for oﬄine analysis.

#### Magnetic Resonance Imaging

Whole brain imaging was conducted using a Siemens 1.5T Avanto Mobile MRI system with advanced SQ gradients (max slew rate 200 T/m/s, 346 T/m/s vector summation, rise time 200 μs) equipped with a 12-element head coil. Functional images were collected (TR = 2 s; TE = 39 ms; flip angle = 75°; FOV = 24 cm × 24 cm; matrix = 64 × 64; in plane resolution = 3.75 mm × 3.75 mm; slice thickness = 5 mm; 27 axial oblique slices) during the experiment. The conditioning run consisted of one hundred fifty whole brain scans. High resolution MPRAGE images (1 mm slices) were collected in a sagittal orientation (flip angle = 8°; FOV = 24 cm) and served as an anatomical map for the functional images.

### Data Analysis

Our primary interest was in examining the relationship between psychopathy and anxiety on the behavioral and neural indices of fear acquisition. To this end we first created CS+ > CS- difference scores for each of the dependent measures described below. We then modeled these dependent measures using a linear mixed effects model treating psychopathy as a categorical variable and anxiety as a continuous variable, which is preferred over an ANOVA because it allows for the combination of categorical and continuous variables, and does not rely on equal sample sizes. This approach allowed us to assess the following: the overall conditioning effect (i.e., the model intercept), the main effect of psychopathy (controlling for anxiety), the main effect of anxiety (i.e., the group-level correlation between anxiety and the CS+ > CS- difference scores), and the psychopathy by anxiety interaction (i.e., the variability in anxiety/difference score correlations as a function of psychopathy).

After finding a relationship between psychopathy and anxiety, we conducted additional hypothesis driven analyses comparing psychopathic subtypes. These analyses treated psychopathy and anxiety as categorical variables consistent with previous research on subtypes of psychopathy. An alpha level of 0.05 was used for all statistical analyses unless otherwise specified.

#### UCS Expectancy

UCS expectancy data was analyzed by calculating the mean expectancy rating from the last 4 s of each CS presentation (Schultz et al., 2012). We then calculated the mean expectancy ratings for CS+ and CS- trials. Finally, the CS- ratings were subtracted from the CS+ ratings to yield a difference score.

#### Skin Conductance

Skin conductance data was converted to a percent change from baseline using the 2 s preceding each stimulus onset as the baseline for each trial. We then identified the peak percent change from baseline during each CS period. The peak CS- response was subtracted from the peak CS+ response to yield a difference score.

#### Functional Magnetic Resonance Imaging

Image processing and reconstruction was completed using AFNI (Cox, 1996) software. Raw data were motion corrected, put through an edge detection algorithm and registered to the fifth volume of the functional run. Data were visually inspected for head movement and images with large, discrete head movements were censored. Subjects with excessive head movement (greater than 2.5 mm displacement or with five or more examples of large discrete movements) were excluded from further analysis. High resolution structural scans were processed with freesurfer (Fischl et al., 2002, 2004a,b) and warped to Talairach space. We used the 3dDeconvolve program in AFNI (Cox, 1996) to calculate the impulse response function (IRF) evoked by the CS+ and the CS-. Head movement and gross motor responses related to the operation of the UCS expectancy measure were included as regressors of no interest. Four images starting 2 s after stimulus onset were used to calculate the percent area under the curve (%AUC). These maps were blurred using a 4 mm full-width at half-maximum Gaussian kernel. We subtracted the CS- response from the CS+ response and used the difference score maps for statistical analysis. We used the AFNI program 3dLME to conduct the linear mixed effects model on the functional magnetic resonance imaging (fMRI) data. Cluster thresholding (Forman et al., 1995), using Monte Carlo simulations with the AFNI program AlphaSim, was used to correct for multiple comparisons in the whole brain analysis (*p* = 0.005; cluster connection radius = 2 mm; volume = 200 mm^3^; corrected *p* < 0.05).

## Results

### Behavior

The results for the behavioral data are reported for a linear mixed effects model with psychopathy as a categorical variable and anxiety as a continuous variable. However, the interaction reported between psychopathy and anxiety was significant when we calculated a 2 (Control, Psychopath) by 2 (High Anxiety, Low Anxiety) ANOVA treating both psychopathy and anxiety as categorical variables.

#### UCS Expectancy

**FIGURE 2:**
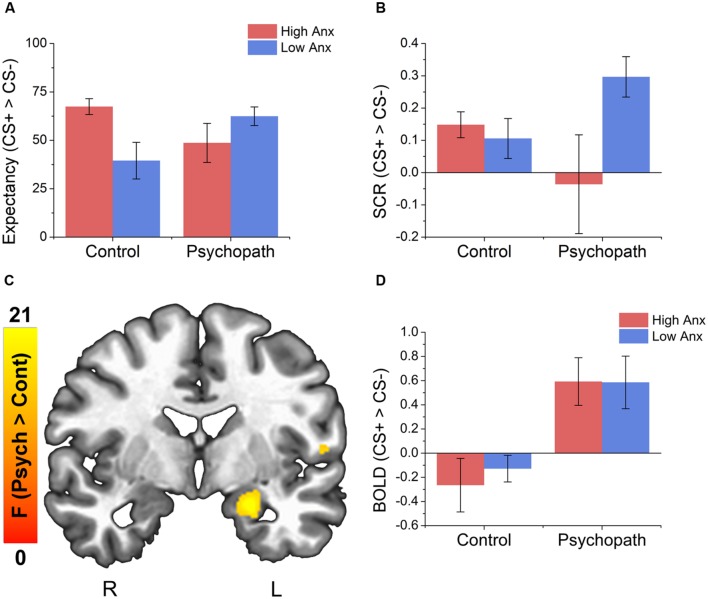
**Behavioral data indicates an interaction between psychopathy and anxiety on both UCS expectancy and SCR measures and psychopaths show larger differential activity in the left amygdala compared to the control group. (A)** All groups give larger UCS expectancy rating for the CS+ relative to the CS-. The control group shows a trend for larger differential UCS expectancy ratings in the high anxiety group compared to the low anxiety group. There was no such trend for the psychopath group. **(B)** Psychopaths in the low anxiety group (primary) show a trend toward a larger differential SCR compared to Psychopaths in the high anxiety group. There was no such trend in the control group. **(C)** Brain map showing larger differential activity in the left amygdala for psychopaths. **(D)** Bar graph of the data from the amygdala cluster. Error bars depict the standard error of the mean. The colors on the brain map correspond to the *F*-values on the color scale.

We analyzed the UCS expectancy difference scores and found that the intercept of the model was significantly greater than 0 [*M* = 53.42; *SEM* = 4.59; *t*(46) = 11.64; *p* < 0.001; Cohen’s *d* = 1.56], suggesting that the sample as a whole learned the stimulus contingencies. In addition, we found a significant psychopathy × anxiety interaction [*t*(46) = 2.38; *p* = 0.02], but no main effects (*ps* > 0.01). To probe the interaction, we split the psychopaths and control subjects into groups based on high and low anxiety as described in Section “Materials and Methods.” In the Control group we found slightly larger differential UCS expectancy in high anxiety individuals compared to low anxiety individuals [*t*(17) = 2.06; *p* = 0.049; Cohen’s *d* = 0.86; **Figure 2A**], but there was no such trend in the Psychopath group (*p* > 0.1). All groups reported larger expectancy ratings on CS+ trials than on CS- trials.

#### Skin Conductance

As with UCS expectancy, we analyzed the SCR difference scores using a linear mixed effects model with psychopathy as a categorical variable and anxiety as a continuous variable. As before, we found that the intercept of the model was significantly greater than 0 [*M* = 0.14; *SEM* = 0.04; *t*(46) = 3.76; *p* < 0.001; Cohen’s *d* = 0.48], suggesting that the sample as a whole showed a differential fear response. We also found a significant psychopathy × anxiety interaction [*t*(46) = 2.19; *p* = 0.03], but no main effects (*ps* > 0.01). When we followed this interaction with *post hoc t*-tests we saw a different pattern of results. Unlike UCS expectancy, we found that Psychopaths with low anxiety (primary psychopaths) showed a somewhat larger differential SCR [*t*(17) = 2.06; *p* = 0.068; Cohen’s *d* = 0.75; **Figure 2B**] than those with high anxiety (secondary psychopaths). This pattern was not seen in the Control group (*p* > 0.1).

### Imaging

As described in Section “Materials and Methods” we identified the BOLD response evoked by the CS+ and the CS- and converted these values to a single CS+ minus CS- difference score. We then analyzed these difference scores across the entire brain using a linear mixed effects model with psychopathy as a categorical variable and anxiety as a continuous variable. We conducted a follow-up analysis as a 2 (Psychopath, Control) by 2 (High Anxiety, Low Anxiety) ANOVA treating psychopathy and anxiety as categorical variables to examine the neural responses in different subtypes of psychopathy as discussed in Section “Materials and Methods.”

### Anxiety as a Continuous Variable

#### Conditioning Main Effects

To test the general effects of conditioning, we examined the activation map corresponding to the intercept of the general linear model (see **Table 2** for a complete list of the results). Like previous conditioning studies with healthy individuals (Cheng et al., 2003; Knight et al., 2004a,b; Milad et al., 2007a) we show activation in a set of regions important for the expression of emotion. These regions include the left amygdala, the left dorsal anterior cingulate cortex (ACC), the left middle frontal gyrus, and several regions of the cerebellum and visual cortex. In all cases, the intercept of the model in these regions was positive, suggesting a CS+ > CS- conditioning effect.

**Table 2:** Conditioning effects.

Structure	Coordinates			*F*	Volume (mm^3^)	Effect
	RL	AP	IS			
*Conditioning main effect (intercept)*						*Intercept (CS+ > CS*−*)^∗^*
Right culmen	–12.9	66.5	–8.9	10.9	2267	0.55
Right superior frontal gyrus/BA6	–7	–11	48	11.3	917	0.47
Left declive	29	61	–19	10.8	809	0.49
Right middle temporal gyrus	–54	60	14	11.0	796	0.57
Left thalamus	0	3	5	14.5	748	0.69
Left culmen	41	45	–28	11.6	565	0.67
Left medial frontal gyrus	1	10	57	11.5	524	0.53
Left precuneus	18	55	50	11.6	519	0.36
Left amygdala	17	9	–14	11.7	461	0.61
Right anterior cingulate/BA24	–5	–15	23	12.2	453	0.55
Right precuneus	–13	57	20	12.2	450	0.36
Left middle frontal gyrus	47	–4	38	13.9	448	0.59
Left culmen	7	40	–1	11.2	407	0.50
Right cuneus/BA17	–6	83	7	11.6	402	0.69
Right culmen	–1	35	–24	13.2	401	0.60
Left culmen	13	59	–19	12.1	380	0.40
Left superior temporal gyrus/BA21	53	44	10	11.1	371	0.44
Right thalamus	–18	23	–2	11.4	370	0.55
Right medial frontal gyrus	–2	–47	12	10.3	352	0.56
Left cuneus	12	73	27	10.7	309	0.36
Left superior temporal gyrus	37	–21	–21	13.3	282	0.99
Left superior temporal gyrus/BA38	26	–12	–29	12.3	248	0.85
Left inferior frontal gyrus/BA47	47	–15	–5	11.2	244	0.90
Left precuneus/BA7	9	47	58	10.9	241	0.55
Right parahippocampal gyrus	–8	35	2	10.4	232	0.67
Left superior frontal gyrus	35	–50	17	10.1	232	0.63
Right inferior frontal gyrus	–40	–15	–15	10.4	229	0.82
Right middle frontal gyrus	–27	7	45	12.2	223	0.35
Left tuber	42	63	–26	11.3	215	1.10
Right posterior cingulate	–7	44	13	11.4	208	0.50

^∗^*Values represent CS+ > CS– difference scores. Coordinates reflect center of mass and appear in Talairach space.*

#### Psychopath versus Control

Interestingly, when we looked at the main effect for psychopathy we saw greater differential activity for psychopaths than controls in a number of areas commonly associated with fear learning (see **Table 3** for a complete list of the results). These included the left amygdala (see **Figures 2C,D**), the right fusiform gyrus, left parahippocampal gyrus, and the right middle frontal gyrus. There were no areas showing greater differential activation for the Control group. The larger differential amygdala response in the Psychopath group was surprising, but given the size (*Volume* = 575 mm^3^), significance (*F* = 13.4), and effect size (Cohen’s *d* = 1.06), we do not feel that our effect is due to a Type I error. The lack of significant differential amygdala activity in the control group was unexpected, so we conducted a follow-up analysis on this group to identify if there was a general deficit in the fear network. This analysis revealed differential activity in a variety of areas in the fear network for the control group including the ACC, visual cortex, medial frontal gyrus, and the inferior frontal gyrus. Thus, the control group does exhibit differential activity in several regions of the fear network, but not in the amygdala. Incarcerated samples have been characterized by less amygdala activity (Kiehl et al., 2001), and that might be a possible explanation for the lack of differential activity in the control groups. Both psychopath groups demonstrated differential amygdala activity despite the incarcerated control group showing less differential amygdala activity.

**Table 3:** Psychopathy × Anxiety Effects.

Structure	Coordinates			*F*	Volume (mm^3^)	Effect
	RL	AP	IS			
*Psychopathy main effect*						*Psychopathy (PSY > CON)^∗^*
Left amygdala	21	6	–15	13.4	575	1.25
Right fusiform gyrus	–30	58	–10	11.6	398	1.03
Left culmen	27	52	–17	10.4	375	1.14
Left parahippocampal gyrus/BA30	12	43	2	11.6	329	1.11
Left declive	25	67	–13	9.9	327	0.96
Right inferior parietal lobule	–34	51	43	11.5	324	0.77
Right caudate	–6	–11	3	11.0	313	1.02
Right superior frontal gyrus	–22	–10	50	10.7	306	0.83
Left precuneus/BA7	14	54	48	12.4	296	0.77
Right cuneus/BA7	–13	72	30	11.7	272	0.87
Right middle frontal gyrus	–43	–5	45	10.6	248	0.96
Left middle temporal gyrus	29	63	19	10.4	217	0.6
Right putamen	–22	–9	–5	11.0	207	0.97
Left precentral gyrus/BA43	50	9	12	10.8	205	0.88
*Anxiety main effect*						*Anxiety coefficient (ANX β)^∗∗^*
Right middle temporal gyrus	–44	2	–34	11.7	464	–0.61
Left nodule	15	56	–30	11.5	280	–0.49
Left middle frontal gyrus	31	–53	3	10.8	237	–0.51
Left post-central gyrus	21	33	58	11.9	224	0.45
Left middle temporal gyrus	42	56	2	12.6	211	–0.41
*Psychopathy* ×*Anxiety Interaction*						*PSY(ANXβ), CON(ANXβ) ^∗∗^*
Left middle frontal gyrus	32	–51	20	12.1	723	(0.57), (–0.61)
Left cingulate gyrus	5	21	24	12.3	444	(0.5), (–0.71)
Right medial frontal gyrus	–8	–34	–12	13.1	348	(0.31), (–0.79)
Left anterior cingulate	1	–54	1	10.7	297	(0.38), (–0.7)
Right caudate	–35	32	2	13.3	290	(–0.36), (0.74)
Right angular gyrus	–42	57	36	13.2	257	(–0.57), (0.76)
Right medial frontal gyrus	–10	16	54	12.8	237	(–0.29), (0.67)
Right thalamus	–18	12	6	11.8	222	(0.6), (–0.5)
Left uncus	11	3	–26	11.8	221	(0.39), (–0.69)

^∗^*Values represent CS+ > CS– difference scores. ^∗∗^Values represent anxiety/BOLD correlation coefficients. Coordinates reflect center of mass and appear in Talairach space. Psychopath (PSY), Control (CON).*

#### High versus Low Anxiety

The main effect for anxiety was largely characterized by a negative correlation between anxiety and differential BOLD responses, suggesting greater CS+ > CS- differences in the low anxiety subjects (**Table 3**). Regions exhibiting this pattern included the left and right temporal gyrus, and the left middle frontal gyrus. One region, the left post-central gyrus was characterized by the opposite pattern, suggesting greater differential responses in individuals with high anxiety.

#### Interaction (Psychopathy × Anxiety)

We found two distinct patterns of interaction (**Table 3**). In regions displaying the first pattern, the psychopath group showed a positive correlation between anxiety and differential BOLD, while the control group showed a negative correlation. Regions showing this pattern include several prefrontal cortical regions including the left middle frontal gyrus, the left cingulate gyrus, and the right medial frontal gyrus. In regions showing the second pattern, the psychopath group showed a negative correlation between anxiety and differential BOLD, while the control group showed a positive correlation. Regions displaying this pattern include the right caudate, the right angular gyrus, and the right medial frontal gyrus.

### Anxiety as a Categorical Variable

**FIGURE 3:**
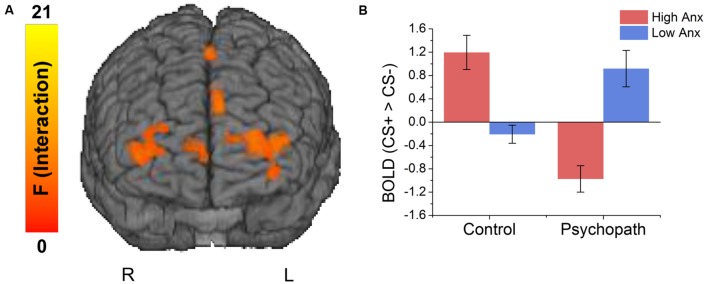
**(A)** Brain regions showing an interaction between psychopathy and anxiety. **(B)** The interaction effects were due to anxiety increasing differential responses to the CS+ versus CS- in the control group, and decreasing differential responses in the psychopaths.

**FIGURE 4:**
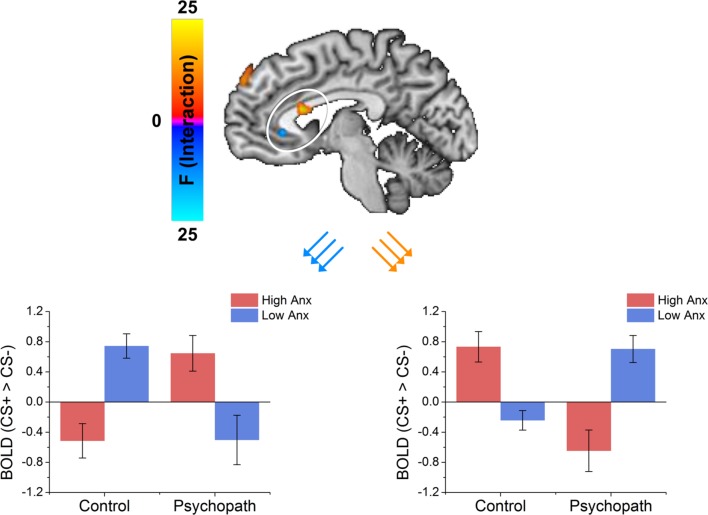
**The dorsal ACC shows an interaction between psychopathy and anxiety characterized by the control group showing that anxiety increased differential responses while the psychopath group shows anxiety decreased differential responses**. The vmPFC shows an interaction between psychopathy and anxiety characterized by the control group showing that anxiety decreased differential responses while the psychopath group shows anxiety increased differential responses.

Because primary and secondary psychopaths showed different behavioral patterns, we wanted to characterize the neural responses related to this behavior. As a follow-up we performed a 2 (Control, Psychopath) by 2 (Low Anxiety, High Anxiety) voxel wise ANOVA on the differential BOLD responses and looked at the regions showing a significant psychopathy by anxiety interaction (see **Table 4** for a complete list of the ANOVA results). In some regions high anxiety in the control group was associated with greater differential CS+ versus CS- responses in, but the high anxiety (secondary) psychopaths showed diminished differential responses relative to the low anxiety (primary) psychopaths. Most regions in the prefrontal cortex showing a significant Psychopathy by Anxiety interaction tended to show this pattern (see **Figure 3**). Among these areas, we saw this pattern bilaterally in the middle frontal gyrus and the dorsal ACC (**Figure 4**), both of which are involved in fear learning and expression (Knight et al., 2004a; Carter et al., 2006; Milad et al., 2007a). In contrast, in other regions high anxiety was related to smaller differential responses in controls while high anxiety (secondary) psychopaths showed greater differential responses relative to low anxiety (primary) psychopaths. Notably this pattern was apparent in the subgenual ACC, an area commonly associated with fear inhibition (Phelps et al., 2004; Milad et al., 2007b; Delgado et al., 2008). The results of treating anxiety as a categorical variable were largely similar to the results we obtained while treating anxiety as a continuous variable. However, distinguishing between these subtypes is important for understanding fear conditioning in psychopathy.

**Table 4:** Psychopathy × Anxiety Group Interaction.

Structure	Coordinates			*F*	Volume (mm^3^)	Effect
	RL	AP	IS			


*Psychopathy* × *Anxiety Interaction*						


Left superior/middle frontal gyrus	29	–58	17	12.1	1822	P(L > H), C(H > L)


Right middle frontal gyrus	–34	–62	17	10.4	1111	P(L > H), C(H > L)


Right middle occipital gyrus	–28	95	12	11.2	853	P(L > H), C(H > L)


Left middle occipital gyrus	18	97	20	11.1	649	P(L > H), C(H > L)


Right precuneus	–15	61	41	10.3	391	P(H > L), C(L > H)


Right inferior parietal lobule	–43	81	24	10.6	353	P(L > H), C(H > L)


Right superior frontal gyrus	–8	–64	16	10.4	345	P(L > H), C(H > L)


Left ACC	4	–15	17	14.3	343	P(L > H), C(H > L)


Left superior frontal gyrus	3	–53	39	11.7	332	P(L > H), C(H > L)


Right thalamus	–18	12	6	11.8	265	P(L > H), C(H > L)


Left subgenual ACC	2	–28	3	13.0	240	P(H > L), C(L > H)


Right angular gyrus	–41	58	34	11.1	240	P(H > L), C(L > H)


Left lateral occipital cortex	48	64	–14	10.2	221	P(H > L), C(L > H)


Right supplementary motor area	–1	–28	61	11.6	209	P(L > H), C(H > L)



*Coordinates reflect center of mass and appear in Talairach space. Psychopath (P), Control (C), High anxiety (H), Low anxiety (L).*

## Discussion

We investigated fear learning in psychopaths compared to well-matched control subjects. We found that primary, but not secondary psychopaths showed robust differential fear conditioning as measured by electrodermal responses. Moreover, we found that this difference in fear learning was accompanied by distinctly different patterns of brain activity. Primary and secondary psychopaths showed inverse patterns of dorsal and ventral ACC activity. Primary psychopaths showed differential electrodermal responses and activation of the dorsal ACC and deactivation of the ventral ACC. These regions have been implicated in previous studies of fear conditioning (Knight et al., 2004b; Carter et al., 2006; Milad et al., 2007a). These results were similar to the high anxiety control group. In contrast, secondary psychopaths showed diminished electrodermal responses and deactivation of the dorsal ACC and activation of the ventral ACC. These regions have been identified in previous studies examining the inhibition of fear (Phelps et al., 2004; Milad et al., 2007b; Delgado et al., 2008). This pattern was similar to the low anxiety control group. Additionally, in contrast to prior work we observed larger differential amygdala responses in psychopaths compared to controls, regardless of level of anxiety. Taken together, these results suggest that the low-fear hypothesis is not sufficient to explain the presence of psychopathy. In fact, these results suggest that the low fear findings often observed in psychopaths, may be driven by secondary psychopathy and they may arise in this group from another factor rather than a deficit in amygdala activity.

Our results are inconsistent with the “low-fear model” of psychopathy. Although, this model has been the foremost explanation for the emergence of psychopathic symptoms, there have been relatively few well-controlled laboratory studies of fear acquisition in psychopaths. Previous studies have used liberal inclusion criteria to identify psychopaths (e.g., use a lower threshold on the PCL-R than the recommended 30; Rothemund et al., 2012), included poorly matched control subjects, or unconventional laboratory fear conditioning procedures (e.g., odor as UCS as opposed to shock; Flor et al., 2002; Birbaumer et al., 2005). Furthermore, there has yet to be a study of fear acquisition in psychopaths that distinguishes between the distinct etiological primary and secondary psychopathy subtypes. This study was a first step in addressing these gaps in the literature. First, we identified psychopathy using the PCL-R checklist, a gold-standard interview-based measure. Second, as recommended by the PCL-R manual, we used a strict cut-off (PCL-R score > 30) to identify psychopaths. Third, we recruited psychopaths and control subjects from the same pool of incarcerated individuals. Fourth, we assessed fear acquisition using procedures that have been repeatedly validated in the literature (Knight et al., 1999, 2004b, 2010; Cheng et al., 2006, 2007; Dunsmoor et al., 2008; Schultz et al., 2012).

In contrast to the low-fear model, some have proposed that deficits in threat processing may be due to abnormal attentional processing (Zeier et al., 2009; Newman et al., 2010). A critical difference between the low-fear and attention models of psychopathy concerns predictions regarding the global versus situation-specific nature of psychopaths’ fear deficit. To the extent that psychopathy involves an absolute amygdala-mediated fear deficit, their insensitivity to threat cues should be apparent regardless of experimental circumstances. However, there is now considerable evidence that their deficit in threat processing is context specific rather than absolute (Dadds et al., 2006; Newman et al., 2010; Baskin-Sommers et al., 2011, 2013; Decety et al., 2013; Meffert et al., 2013). Across a variety of studies using passive-avoidance learning, electrodermal responses to threat stimuli, fear potentiated startle and amygdala activity to assess fear, psychopathic offenders display fear deficits when threat cues are peripheral to their primary focus of attention but normal fear responses to centrally presented (i.e., focal) threat cues (Larson et al., 2013). Thus, the fact that psychopaths, specifically primary psychopaths, displayed normal fear conditioning to centrally presented (i.e., focal) CSs may appear anomalous from the low-fear perspective but is congruent with predictions generated by attention-based models of psychopathy (Patterson and Newman, 1993; Baskin-Sommers et al., 2011).

Despite the central presentation of conditioned stimuli in this study, there was some evidence that high-anxious, secondary psychopaths displayed a fear deficit. Thus, the attention-based models do not easily explain this deficit. Although, we did not predict this finding, it is noteworthy that it parallels earlier evidence that the smaller electrodermal responses to threat cues displayed by psychopathic offenders were specific to high-anxious psychopaths (Arnett et al., 1997). Such findings suggest that the higher levels of anxiety associated with this group undermine fear responses, despite the fact that equally high levels of anxiety are associated with normal fear responses in high-anxious non-psychopaths. Thus, anxiety appears to interact with level of psychopathy to augment or undermine fear. Another study found that disruption of the dopamine system during conditioning can result in a decrease in electrodermal responses, but not on a measure of anxiety (Menon et al., 2007), which was interpreted as a deficit in responding to salient cues. The brain areas most associated with psychopathy by anxiety interactions in the current study involved ventral and dorsal ACC, which are key nodes in circuitry instantiating inhibition of negative affect (vACC; Phelps et al., 2004; Roy et al., 2012), expression of conditioned fear (dACC; Milad et al., 2007a), and integration of negative affect and cognitive control (dACC; Shackman et al., 2011). Thus, among psychopaths, anxiety may increase attempts to regulate fear. More generally, our findings suggest that secondary psychopathy may be at least in part responsible for prior findings of impaired fear conditioning in psychopathy. Future research is needed to further evaluate this possibility and better characterize how anxiety influences cognitive control and affect regulation circuitry in psychopaths.

One of the strengths of this study was we used stringent inclusion criteria for inclusion in the psychopathy group, and that we compared these individuals to a well-matched control group from the same prison facility. However, this resulted in relatively small sample sizes in our groups. However, it should be noted that we were able to show robust conditioning in the primary psychopaths, and cell sizes were comparable to previous studies of fear conditioning in psychopathy.

In this study we measured conditioned fear acquisition and fMRI in primary and secondary psychopaths, and matched controls. In contrast to the low-fear hypothesis, we did not see a general deficit in fear expression in psychopaths. Instead we saw typical fear expression in primary psychopaths, and reduced fear expression in secondary psychopaths. These behavioral results were accompanied by an increased fear response in the amygdala for psychopaths. We also observed inverse patterns of ACC activity for primary and secondary psychopaths. Primary psychopaths showed a pattern of dorsal and ventral ACC expression, while secondary psychopaths showed a pattern consistent with fear inhibition. These results suggest that the low fear observed for psychopaths in previous conditioning studies may be carried by secondary psychopaths.

## Author Contributions

DS helped design, collect, analyze, interpret, and write the manuscript. NB helped design, collect, analyze, interpret, and write the manuscript. AB-S helped interpret results and write the manuscript. CL helped design, interpret, and write the manuscript. FH helped design, collect, interpret, and write the manuscript.

## Conflict of Interest Statement

The authors declare that the research was conducted in the absence of any commercial or financial relationships that could be construed as a potential conflict of interest.
