# Corrigendum: Psychopaths Show Enhanced Amygdala Activation during Fear Conditioning

**DOI:** 10.3389/fpsyg.2017.01457

**Published:** 2017-08-22

**Authors:** Douglas H. Schultz, Nicholas L. Balderston, Arielle R. Baskin-Sommers, Christine L. Larson, Fred J. Helmstetter

**Affiliations:** ^1^Department of Psychology, University of Wisconsin-Milwaukee Milwaukee, WI, United States; ^2^Department of Psychology, Yale University New Haven, CT, United States; ^3^Department of Neurology, Medical College of Wisconsin Milwaukee, WI, United States

**Keywords:** psychopathy, fear conditioning, anxiety, amygdala, fMRI

There was a mistake in one of the papers referenced as published. Originally we cited: Hare, R.D., and Vertommen, H. (2003). The Hare Psychopathy Checklist-Revised. Toronto, ON: Multi-Health Systems.

This citation is inaccurate. The reference should appear as:

Hare, R. D. (2003). The Hare Psychopathy Checklist-Revised, 2nd Edn. Toronto, ON: Multi-Health Systems.

Additionally, the paper is cited in the text multiple times on page 2. The in-text citations should appear as Hare (2003).

The authors apologize for the mistake. This error does not change the scientific conclusions of the article in any way.

## Conflict of interest statement

The authors declare that the research was conducted in the absence of any commercial or financial relationships that could be construed as a potential conflict of interest.
